# Dose Assessment in Computed Tomography Examination and Establishment of Local Diagnostic Reference Levels in Mazandaran, Iran

**Published:** 2015-12-01

**Authors:** A. Janbabanezhad Toori, A. Shabestani-Monfared, M.R. Deevband, R. Abdi, M. Nabahati

**Affiliations:** 1 School of Medicine, Babol University of Medical Sciences, Babol, Iran; 2 Department of Medical Physics, Faculty of Medicine, Babol University of Medical Sciences, Babol, Iran; 3 Department of Medical Physics, School of Medicine, Shahid Beheshti University of Medical Sciences, Tehran, Iran; 4 Department of Radiology, Mazandaran University of Medical Sciences, Sari, Iran; 5 Department of Radiology, Babol University of Medical Sciences, Babol, Iran

**Keywords:** Diagnostic Reference Levels, Computed Tomography, Mazandaran, CTDI, DLP

## Abstract

**Background:**

Medical X-rays are the largest man-made source of public exposure to ionizing radiation. While the benefits of Computed Tomography (CT) are well known in accurate diagnosis, those benefits are not risk-free. CT is a device with higher patient dose in comparison with other conventional radiation procedures.

**Objective:**

This study is aimed at evaluating radiation dose to patients from Computed Tomography (CT) examination in Mazandaran hospitals and defining diagnostic reference level (DRL).

**Methods:**

Patient-related data on CT protocol for four common CT examinations including brain, sinus, chest and abdomen & pelvic were collected. In each center, Computed Tomography Dose Index (CTDI) measurements were performed using pencil ionization chamber and CT dosimetry phantom according to AAPM report No. 96 for those techniques. Then, Weighted Computed Tomography Dose Index (CTDIW), Volume Computed Tomography Dose Index (CTDI vol) and Dose Length Product (DLP) were calculated.

**Results:**

The CTDIw for brain, sinus, chest and abdomen & pelvic ranged (15.6-73), (3.8-25. 8), (4.5-16.3) and (7-16.3), respectively. Values of DLP had a range of (197.4-981), (41.8-184), (131-342.3) and (283.6-486) for brain, sinus, chest and abdomen & pelvic, respectively. The 3rd quartile of CTDIW, derived from dose distribution for each examination is the proposed quantity for DRL. The DRLs of brain, sinus, chest and abdomen & pelvic are measured 59.5, 17, 7.8 and 11 mGy, respectively.

**Conclusion:**

Results of this study demonstrated large scales of dose for the same examination among different centers. For all examinations, our values were lower than international reference doses.

## Introduction

Nowadays, x-ray plays an important role in medical decisions and, in some cases, early detection of diseases is possible solely through x-ray examinations[1]. Although x-ray is a very useful and essential tool in healthcare and has numerous advantages for human societies, it is also known as a carcinogenic agent[2]. Along with increasing development in medical imaging systems and techniques, radiation of patients is increasing and now medical x-ray is responsible for most radiation to population from artificial sources[3]. Computed Tomography (CT) is an x-ray imaging modality with high radiation dose (10-100 times greater than conventional x-ray)[4]. Since 1972, when CT emerged, use of this imaging procedure has been increased and today the most detectable part of medical ionizing radiation to population is caused by CT examinations[5]. Based on European Commission’s data, in some countries, 40% of medical radiation is due to CT[6]. National Radiation Protection Board (NRBP) showed that in 2003, CT was responsible for 47% of medical radiation of the UK population[7]. In CT examination, the probability of radiation-induced cancer is more than other x-ray examinations because of high level of radiation dose (10-100 mGy) imparted in tissues[8]. Many studies revealed large differences in radiation doses for the same CT examination among different hospitals and these variations are due to different examination techniques and CT scanner models[6, 9]. There is an essential need for establishing a reference level of activity with the aims of comparing different techniques and protocols to find situations where examination procedures must be reviewed. In 1996, International Commission on Radiological Protection in ICRP Publication 73 introduced Diagnostic Reference Level (DRL) for optimization of patient-radiation protection in medical Examinations[10]. The aim of DRL is to reduce patient-radiation dose without affecting medical diagnosis. Establishment and use of DRL is a method to identify situations where the level of patient-radiation dose is unnecessarily high. DRL can suggest applicable approaches to reducing patient dose to an acceptable level. Hatziioannou et al[11] established Greece national DRL for six typical CT examinations including brain, cervical spine, chest, abdomen, lumbar spine and pelvic. Their study was performed on 27 CT systems and the results showed large inter-center variation in dose of the same examinations. Treier et al[12] also examined 21 CT examinations on 179 scanners. Their study was performed on adult and pediatric-age groups and new DRLs were set for Switzerland. There is no study on patient dose in CT exams in Mazandaran. This study is trying to make an appropriate quantity of radiation dose as a DRL for CT hoping that the results can improve radiation protection procedures in CT examinations.

## Materials and Methods

In CT dosimetry, because of the particular geometry of the device and irradiation, we need to define a proper dose descriptor[11]. Computed Tomography Dose Index (CTDI) is considered as dose descriptor in CT. Weighted Computed Tomography Dose Index (CTDI_w_) is the first proposed quantity as a reference dose for a single axial rotation:

$$CTDIw=\frac{1}{3}CTDIc+\frac{2}{3}CTDIp\;\left(\text{mGy}\right)\;(1)$$

where CTDI_c_ is the dose in the central hole and CTDI_p_ is the mean dose of four periphery holes of phantom.

In spiral mode, volume CTDI (CTDI_vol_) is calculated:

$$CTDIvol=\frac{CTDIw}{Pitch}\;\left(\text{mGy}\right)\;(2)$$

where, pitch is the ratio between table increment per rotation and beam width[13-15].

Another reference quantity is Dose Length Product (DLP) that expresses total dose in a complete examination:

$$DLP=CTDIw\times N\times T\;\left(\text{mGy}\right)\;(3)$$

where N is the number of slices and T is the slice thickness. If examination is performed in helical mode, DLP is calculated as following equation:

$$DLP=CDTIvol\times L\;\left(\text{mGy}\right)\;(4)$$

where L is the scan length. 

**Figure 1:**
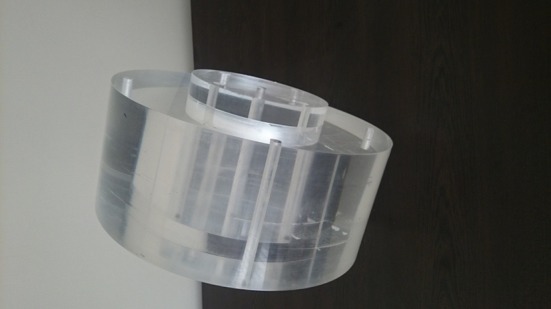
Head and body CT dosimetry phantom

This survey was performed in seven public hospitals in Mazandaran, Iran. Four CT examinations with highest percentage of total examination including brain, sinus, chest and abdomen & pelvic were selected for this study. Questionnaires were developed to collect primary data on patients, CT systems (type, manufacturer, number of detector rows) and protocols (kvp, mAs, slice thickness, number of slices, pitch and table increment). In all centers, the information of ten typical patients was recorded for each exam. For CTDI measurement, Barracuda dosimetry kit (Barracuda X-ray Analyzer, RTI Electronics, Sweden) was used. This kit includes a pencil ionization chamber with serial number of 1673 which has an active length of 10cm and two CT dosimetry phantoms. For dosimetry in medical X-ray imaging system, ionization chamber system and TLD system are most commonly used. Suitable ionization chamber has known advantages over TLD systems in that their accuracy, precision, and energy independence are better. In addition, ionization chambers can be read out directly. Two cylindrical polymethylmetacrylate phantoms with different diameters were used as patients’ representatives (figure 1).

Head phantom, with 16cm diameter and 15cm length, has five holes; one in the center and four located 1cm under the surface making 90-degree angle altogether. Body phantom covers 32cm diameter and 15cm length and is similar to head phantom. For the measurement of CTDI_C_, head phantom was placed in bed head holder, located in iso-center. Then, ionization chamber was placed in central dosimetry hole and other holes were filled with PMMA plugs. According to questionnaire, a single axial scan was selected and the dose was recorded. To calculate CTDI_P_, this procedure was repeated for four peripheral holes. Dose measurement in body phantom was performed in the same way when it was positioned on the table. Then, the CTDI_W_ and DLP were calculated for all examinations using the previously mentioned method.

## Results

**Figure 2:**
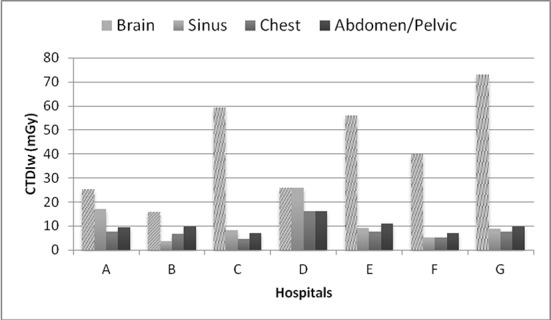
Calculated CTDI_w_(mGy) of all examination in different hospitals

**Figure 3:**
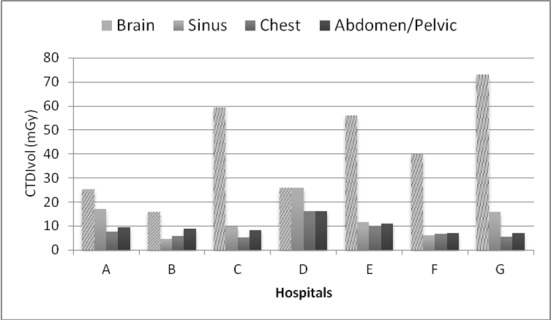
Calculated CTDI_vol_(mGy) of all examination in different hospitals

**Figure 4:**
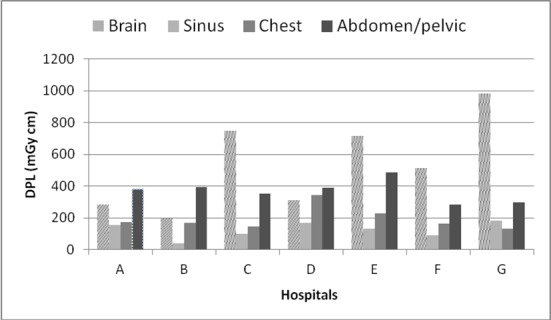
Calculated DLP (mGy cm) of all examination in different hospitals

**Figure 5:**
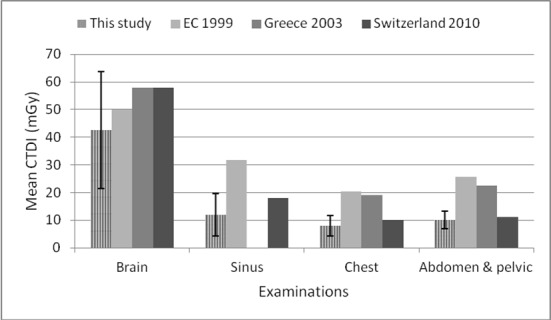
Mean CTDI value of this study in comparison with the national and international proposed values. Error bars represent the standard deviation. Greece study did not include sinus examination.

Table 1 presents protocol details for all examinations used in seven hospitals. As shown in table 1, scan parameters for the same examinations are different from hospital to hospital. Although tube voltages (kvp) are nearly similar, variations in tube current (mAs) are significant. For example, kvp of brain examination in two centers B and G are identical but mAs in center G is 3.8 times greater than that of center B. This factor probably is the main reason of higher radiation dose in hospital G. Similar discrepancies are observed for three other examinations as well. Scan length is also different among hospitals especially in chest examinations (ranging 23-30 cm). Figure2, 3 and 4 show the calculated CTDI_W_, CTDI_vol_ and DLP for each examination in different hospitals. As can be seen, there are large Scales in CTDI_W_, CTDI_vol_ and DLP values I different hospitals. CTDI_vol_ values in this study are nearly similar to CTDI_w_ (see figures 2 and 3). This was due to the use of pitch factor of 1 or close to 1 (0.85 to 1.11) in most hospitals. The exception was sinus examination in hospital G, where the pitch was 0.562 and CTDI_vol_ was higher than CTDI_w_ (16.01 vs 9). The mean, range and standard deviation of calculated CTDI_W_ and DLP are given in table 2. Figure 5 compares mean value of our CTDI in comparison with some European countries. As can be seen, mean values of Mazandaran CTDI for all examinations are lower than international values. However, large error bars representing standard deviation showed that dose of same scan areas are significantly different among these hospitals. As shown in table 2, CTDI_W_ had a range of (15.6-73), (3.8-25.8), (4.5-16.3) and (7-16.3) for brain, sinus, chest and abdomen & pelvic, respectively. It is internationally accepted that the 3rd quartile of dose distribution is considered as reference dose. Therefore, according to these results, values 59.5, 17, 7.8 and 11 mGy are proposed as DRL for brain, sinus, chest and abdomen & pelvic, respectively. Table 3 shows our DRL in comparison with international proposed reference doses. As shown in table 3, our DRLs for all examinations were lower than international values. CTDI values in CT procedures are related to exposure parameters including mAS and kVp. In addition, DLP is increased by elevating the number of slices and scan length. Therefore, DLPs in abdomen and chest examinations are higher than head examinations. On the other hand, DLP and CTDI increase as size goes up. The mean CTDIw  and DLP values in Mazandaran were below in comparison with European Guidelines (EG) and Shrimpton et al.

**Table 1:** Protocol information used in different hospitals

**Examination**	**protocol**	**Hospitals**						
		A	B	C	D	E	F	G
Brain	kvp	120	110	130	120	130	110	110
	mAs	170	100	250	140	270	270	380
	L(cm)	12	12.6	12.6	12	12	13.4	12.8
Sinus	kvp	120	110	130	120	130	110	100
	mAs	112	35	35	140	35	35	60
	L(cm)	9	11	10.4	9	13.3	13.7	11.5
Chest	kvp	120	130	130	120	130	110	100
	mAs	100	70	70	140	70	70	105
	L(cm)	21	29	27	21	30	24.4	23.6
Abdomen & pelvic	kvp	120	130	130	120	130	110	100
	mAs	130	120	120	224	120	120	105
	L(cm)	40	44	42.4	38	44.3	40.5	42

**Table 2:** Mean, range and Standard deviation of CTDI_W_ (mGy) and DLP (mGy cm) for four typical CT examinations

**Examination**	**CTDIw (mGy)**			**DLP (mGy)**		
	Mean	Range	St.deviation	Mean	Range	St.deviation
Brain	42.16	15.8-73	21.2	535.6	194.4-981	291
Sinus	11.19	3.8-25.8	7.68	124	41.8-167.7	49.8
Chest	7.94	4.51-16.3	3.8	193	131-342	72.8
Abdomen & pelvic	10	7-16.3	3.12	393.3	283.6-486	67.9

**Table 3:** The 3rd quartile values of this study compared with international values

**Examination**	**This study**		** EC[6] **		** Shrimpton et al[7] **	
	CDTI_w_	DLP	CDTI_w_	DLP	CDTI_w_	DLP
Brain	59. 5	750	60	1050	66	787
Sinus	17	167	35	360	-	-
Chest	7. 8	230	30	650	17	488
Abdomen & pelvic	11	395	35	780	19	472

In general, Mazandaran DRLs for all examinations were lower than international values.  It is recommended that this reference dose be temporarily considered as standard dose for optimization procedures until further studies are conducted and information on all CT examinations collected. 

## Discussion

This was the first comprehensive study on patient doses in CT examinations performed in Mazandaran, Iran and the DRL was set for four CT examinations. As mentioned under Results section, doses for the same examinations varied from hospital to hospital. Probably, this discrepancy was due to different types of protocols, user selection parameters (such as kvp, mAs, pitch and slice thickness) and differences in the design of CT devices by the manufacturers. There are studies conducted in Iran and some reference doses were proposed. Toossi presented values of 58.08, 28.31 and 28.31 as Iranian national DRL for brain, chest and abdominal examinations, respectively[9]. Their values were higher than those in the current study except for the brain. Afzalipour et al[9] presented Local DRL (LDRL) for the same examinations in Tehran province. CTDI_W_ of their study for brain (50.78 mGy) and abdomen (9.11 mGy) was lower than our values. For DLP, except for the sinus (167 vs. 210.46 mGy cm), our values were higher and the largest differences are found in brain examinations (750 vs. 422.64 mGy cm). In some European countries, national DRLs for adults have been established[6, 7, 11, 12]. For brain examination, the greatest differences for DRLs of CTDI observed between Mazandaran, Iran and Greece (59.5 vs. 69.9) and for DRLs of DLP between Mazandaran, Iran and EC (750 vs. 1050). For other examinations, the largest differences are also found between Mazandaran, Iran and EC (table 3). Although our recommended DRL is lower than international references, large extent of dose distribution particularly in brain examination indicates that CT procedures in Mazandaran province are required to be optimized. In brain examination, hospital G had the highest CTDI_W_ and CTDI_vol_ exceeding international recommended values. This was probably caused by high level of mAs used in this hospital. A practical way to optimize the radiation dose is reducing mAs, provided that medical diagnosis is not affected. CTDIW and CTDI_vol_ values of other examinations in all centers were lower than those recommended by international organizations. In all examinations, DLP values had significant differences among hospitals. For example, in brain examination, DLP of center G stood 4.9 times greater than center B and in abdomen and pelvic, center E was 1.7 times greater than center F. This was because of the differences in CTDI_W_ and scan length among hospitals. As shown in table 1, center E had the highest scan length for abdomen & pelvic examinations. Scan length has significant impact on patient dose and should be limited to areas which help the diagnosis process. There are some other approaches for dose reduction such as decreasing kvp, increasing pitch factor and the use of Automatic Exposure Control (AEC). Staff training and their awareness of technical parameters of CT examination also have significant effects on patient’s dose. The results of this study can be introduced to hospitals and CT users to become aware of their activities and also protocols used in other centers. Dose measurement should be performed after appropriate period of time and compared with current study. In this study, due to time constraints, only four CT examinations were evaluated. Therefore, additional research on other CT examinations is required to determine reference doses for all CT exams.
